# The association between genetic polymorphisms of the interleukin-23 receptor gene and susceptibility to uveitis: a meta-analysis

**DOI:** 10.1186/s12886-017-0477-4

**Published:** 2017-05-30

**Authors:** Jae Hyun Jung, Gwan Gyu Song, Jae-Hoon Kim, Young Ho Seo, Sung Jae Choi

**Affiliations:** 1 Korea University College of Medicine, Seoul, South Korea; 2 Department of Rheumatology, Korea University Guro Hospital, 148, Gurodong-ro, Guro-gu, Seoul, 08308 South Korea; 3 Division of Rheumatology, Department of Internal Medicine, Korea University Ansan Hospital, 123, Jeokgeum-ro, Danwon-gu, Ansan-si, Gyeonggi-do 15355 South Korea

**Keywords:** Interleukin-23R, Polymorphism, Uveitis, Meta-analysis

## Abstract

**Background:**

Uveitis is an eye inflammatory disease, which is sometimes associated with underlying systemic disease. Interleukin-23 plays an important role in autoimmune disease. The aim of this meta-analysis was to evaluate the association between the interleukin-23 receptor (IL-23R) and susceptibility to uveitis.

**Methods:**

Published literature from PUBMED and EMBASE were retrieved. Seven studies were included in this meta-analysis, covering a total of 1309 cases of uveitis and 2400 controls. Meta-analyses were conducted on the associations between uveitisand rs7517847, rs17375018, and rs11209032 polymorphisms in the IL-23R gene.

**Results:**

There were no significant associations between IL-23R polymorphisms and uveitis with regard to the following alleles: for G allele vs. T allele of rs7517847, OR 1.01, 95% CI 0.92–1.12, *P* = 0.83; for A allele vs. G allele of rs17375018, OR 0.68, 95% CI 0.47–0.99, *P* = 0.05; rs11209032 OR 1.12, 95% CI 0.84–1.51, *P* = 0.43. In contrast, there were significant associations between the AA + AG gene versus GG gene of rs17375018 and the AA gene versus AG + GG gene of rs11209032 polymorphism with uveitis (OR 0.59, 95% CI 0.35–0.99, *P* = 0.04; OR 1.32, 95% CI 1.10–1.59, *P* = 0.003).

**Conclusions:**

This meta-analysis suggests that each allele of IL-23R, including rs7519847, rs17375018 and rs11209032 was negatively associated with uveitis. However, homozygote models, including the rs17375018 GG genotype and rs11209032 AA genotype, were significantly associated with uveitis.

## Background

Uveitis, or inflammation of the uvea, is classified clinically into infectious and non-infectious groups [1]. Infectious uveitis is triggered by a wide range of exogenous factors. In contrast, non-infectious uveitis is an inflammatory response that is triggered by certain environmental factors in individuals with a particular genetic component. Non-infectious uveitis is an autoimmune process by which there is a loss of tolerance against self-antigens. Certain HLA alleles have been found to be strongly associated with uveitis [2].

In up to 50% of the cases, autoimmune uveitis precedes or follows the onset of an autoimmune disease. Some of the associated autoimmune diseases include the spondyloarthritides, Behçet’s disease, Vogt-Koyanagi-Harada (VKH) syndrome, systemic lupus erythematosus, sarcoidosis, autoimmune hepatitis, and multiple sclerosis [3]. There is ample evidence that most autoimmune disease share a certain percentage of their genetic component, suggesting that some pathologies may be influenced by a common pathway. The interleukin-23 receptor (IL-23R) belongs to a well-established group of risk factors (shared by different conditions) that influence the breakdown of self-tolerance [4].

The IL-23R gene is located on chromosome 1p31. It is highly expressed in dendritic cells, and is involved in several chronic inflammatory diseases [5]. Several single-nucleotide polymorphisms (SNPs) in IL-23R have been found to be associated with chronic inflammatory diseases, including inflammatory bowel disease, ankylosing spondylitis, and psoriasis. Recent studies found that IL-23R gene polymorphisms are associated with uveitis in Behçet’s disease and sarcoidosis [6, 7]. However, other studies found no significant association between uveitis and IL-23R gene polymorphisms [5, 8].

Three of several polymorphisms of IL-23R have been studied in some detail. These include rs7517847, rs17375018, and rs11209032. However, the results from different studies have been inconsistent. The aim of this meta-analysis was to investigate the genetic association between IL-23R polymorphisms and the susceptibility to non-infectious uveitis.

## Methods

### Identification of eligible studies and data extraction

We performed a search for studies that examined the associations between IL-23R polymorphisms and uveitis. Genetic association studies that determined the distributions of rs7517847, rs17375018, and rs11209032 polymorphisms in uveitis and in normal controls were included. The literature was searched using the PUBMED and EMBASE databases to identify available articles in which IL-23R polymorphisms were analyzed in uveitis patients (up to June 2016). We listed combinations of key words and subject terms such as “interleukin-23 receptor,” “IL23R,” “polymorphism,” and “uveitis.” Only papers written in the English language were included. References from the identified studies were also investigated to identify additional studies that were not indexed by PUBMED and EMBASE. The following information was extracted from each study: author, year of publication, ethnicity of the study population, demographics, number of cases and controls, Hardy-Weinberg equilibrium (HWE) *P*-value, and the allele and genotype frequencies for each of the polymorphisms (rs7517847, rs17375018, and rs11209032). This meta-analysis was reported based on the Preferred Reporting Items for Systemic Reviews and Meta-Analyses (PRISMA) guidelines [9].

### Evaluation of statistical associations

The allele counting method was used to determine the allele frequencies of the genetic polymorphisms. The Chi-squared test was used to detect if the controls in each study conformed to HWE. The associations between rs7517847, rs17375018, rs11209032 and uveitis were estimated using the crude odds ratio (OR) and 95% confidence interval (CI). We performed meta-analyses using the 1) allelic contrast, 2) homozygote contrast, 3) recessive, and 4) dominant models. Inter-study heterogeneity was assessed with the Cochran Q test (in which *P*-value < 0.10 was considered statistically significant heterogeneity) and I^2^ statistics (I^2^ < 25 = no heterogeneity; 25 ≤ I^2^ < 50 = moderate heterogeneity; 50 ≤ I^2^ < 75 = large heterogeneity; 75 ≤ I^2^ < 100 = extreme heterogeneity) [10]. If there were no significance between the study heterogeneity, a fixed-effects model was used [11]. Otherwise, a random-effect model was used [12]. Forest plots were drawn to visualize the overall effect. Meta-analysis was performed using Review Manager software, version 5.3.

### Evaluation of publication bias

Funnel plots are often used to detect publication bias. However, they require a range of studies of varying sizes and subjective judgments. Therefore, we evaluated publication bias using Egger’s linear regression test [13], which measures funnel plot asymmetry on a natural logarithm scale of odds ratios (ORs).

## Results

### Studies included in the meta-analysis

**Fig. 1:**
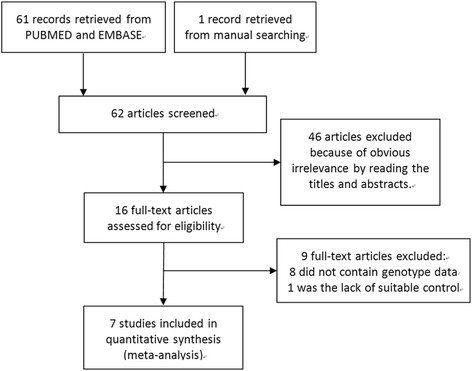
Flow chart of describing the study inclusion/exclusion

Sixty two studies were identified by electronic and manual researches, and 16 were selected for a full-text review based on the title and abstract details. Nine studies were excluded because they did not contain genotype data or were the lack of suitable controls. Ultimately, seven studies met our inclusion criteria [2, 5–8, 14]. A flow chart describing inclusion/exclusion of the individual studies is displayed as Fig. 1. Six of these studies involved the association between the IL-23R gene rs7517847 polymorphism and uveitis, five studies involved rs17375018, and five studies involved rs11209032. A total of seventeen separate comparisons were considered in this meta-analysis, involving 1309 uveitis patients and 2400 controls.

Uveitis in ankylosing spondylitis, Behçet’s disease, sarcoidosis, endogenous non anterior uveitis, VKH, and Fuchs’ syndrome were included in this meta-analysis. Among these diseases, the etiology of Fuchs’ syndrome is not fully understood. Some theories on the etiology of Fuchs’ syndrome have been suggested including infection, vascular abnormality and autoimmunity [5, 15]. Thus, we performed a meta-analysis with and without Fuchs’ syndrome. The details of the IL-23R polymorphism studies are summarized in Table 1.

**Table 1:** Characteristics of the individual studies included in the systemic review and meta-analysis

Study	Population	Numbers		Disease	Gene polymorphism	HWE *P*-value
		Case	Control			
Cenit, 2013 [2]	Spanish (European)	206	1547	Endogenous non anterior uveitis	rs7517847	0.4008
Dong, 2013 [8]	Chinese Han (Asian)	163	312	Ankylosing spondylitis	rs7517847	0.9262
					rs17375018	0.0003
					rs11209032	0.1651
Gheita, 2015 [14]	Egyptian (African)	24	30	Behçet’s disease	rs17375018	0.1221
Jiang(1), 2010 [6]	Chinese Han (Asian)	338	407	Behçet’s disease	rs7517847	0.9014
					rs17375018	0.1697
					rs11209032	0.3495
Jiang(2), 2010 [9]	Chinese Han (Asian)	382	407	Vogt-Koyanagi-Harada syndrome	rs7517847	0.9014
					rs17375018	0.1697
					rs11209032	0.3495
Kim, 2011 [7]	American (American)	58	104	Sarcoidosis	rs7517847	0.9728
					rs11209032	0.9226
Zhou, 2010 [5]	Chinese Han (Asian)	138	407	Fuchs’ syndrome	rs7517847	0.9014
					rs17375018	0.1697
					rs11209032	0.3495

*HWE* Hardy-Weinberg equilibrium

### Meta-analysis of the relationship between IL-23R polymorphisms and overall uveitis

**Fig. 2:**
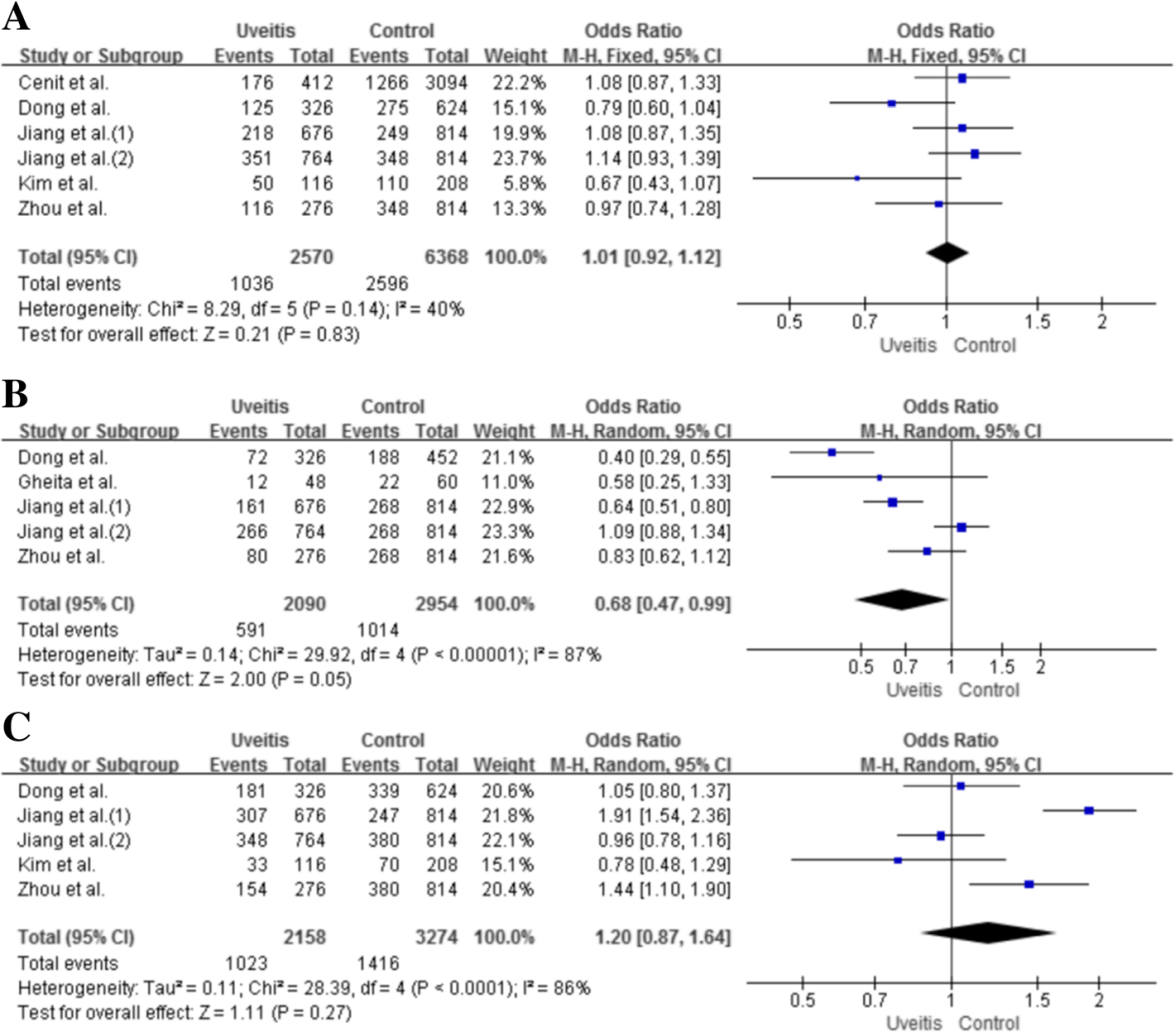
ORs and 95% CIs of the individual studies and of pooled data for the associations between the rs7517847 G allele (**a**), rs17375018 A allele (**b**), rs11209032 A allele (**c**) polymorphisms and overall uveitis

**Fig. 3:**
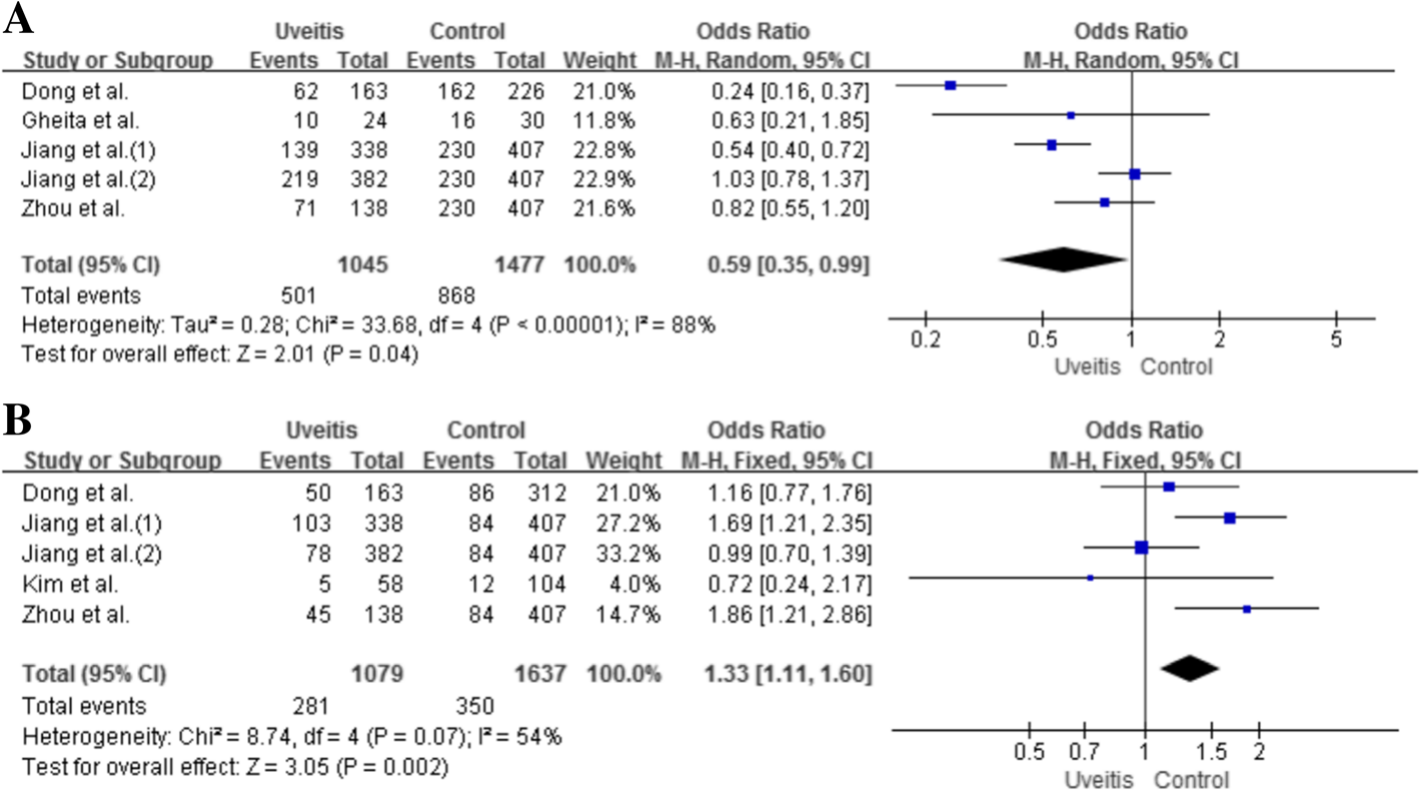
ORs and 95% CIs of the IL23R polymorphism genotype and overall uveitis. rs17375018 AA + AG vs. GG genotype (**a**), and rs11209032 AA vs. AG + GG genotype (**b**)

Table 2 and Fig. 2 demonstrate the findings from our meta-analysis with regard to the association between IL-23R polymorphisms and overall uveitis. There were no differences concerning the genotype and allele of rs7517847 SNPs between patients with uveitis and controls (OR 1.01, 95% CI 0.92–1.12, *P* = 0.83). There was also no significant association between the rs17375018 A allele and uveitis (OR 0.68, 95% CI 0.47–0.99, *P* = 0.05; Fig. 3a). However, the frequency of the rs17375018 AA + AG genotype was significantly associated with uveitis (OR 0.59, 95% CI 0.35–0.99, *P* = 0.04). The allele frequencies of rs11209032 in patients and in controls were not significantly different (OR 1.20, 95% CI 0.87–1.64, *P* = 0.27). A meta-analysis of the AA genotype of the rs11209032 polymorphism was significantly associated with uveitis (OR 1.33, 95% CI 1.11–1.60, *P* = 0.002; Fig. 3b).

**Table 2:** Meta-analysis of associations between IL23R polymorphisms and overall uveitis

Polymorphism		Test of association			Test of heterogeneity		
		OR	95% CI	*P*-value	Model	*P*-value	I^2^(%)
rs7517847	G vs. T	1.01	0.92–1.12	0.83	F	0.14	40
	GG vs. GT + TT	0.70	0.40–1.21	0.20	R	0.00003	81
	GG + GT vs. TT	0.92	0.80–1.07	0.29	F	0.20	32
	GG vs. TT	0.88	0.65–1.19	0.41	R	0.06	53
rs17375018	A vs. G	0.68	0.47–0.99	0.05	R	<0.00001	87
	AA vs. AG + GG	0.76	0.49–1.18	0.22	R	0.09	50
	AA + AG vs. GG	0.59	0.35–0.99	0.04	R	<0.00001	88
	AA vs. GG	0.56	0.29–1.09	0.09	R	0.003	75
rs11209032	A vs. G	1.20	0.87–1.64	0.27	R	<0.0001	86
	AA vs. AG + GG	1.33	1.11–1.60	0.002	F	0.07	54
	AA + AG vs. GG	1.13	0.82–1.56	0.45	R	0.03	64
	AA vs. GG	1.34	0.85–2.12	0.20	R	0.007	71

*R* random effects model, *F* fixed effects model

### Meta-analysis of the relationship between IL-23R polymorphisms and uveitis without Fuchs’ syndrome

**Fig. 4:**
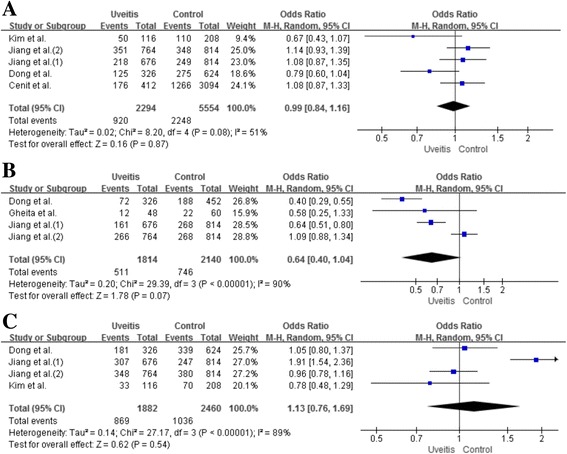
ORs and 95% CIs of the individual studies and of pooled data for the associations between the rs7517847 G allele (**a**), rs17375018 A allele (**b**), rs11209032 A allele (**c**) polymorphisms and uveitis without Fuchs’ syndrome

Table 3 and Fig. 4 demonstrate the findings from our meta-analysis with regard to the association between IL-23R polymorphisms and uveitis without Fuchs’ syndrome. There were no differences concerning the genotype and allele of rs7517847, rs17375018 and rs11209032 SNPs between patients with uveitis and controls (OR 0.99, 95% CI 0.84–1.16, *P* = 0.87; OR 0.64, 95% CI 0.40–1.04, *P* = 0.07; OR 1.13, 95% CI 0.76–1.69, *P* = 0.54, respectively).

**Table 3:** Meta-analysis of associations between IL23R polymorphisms and uveitis without Fuchs’ syndrome

Polymorphism		Test of association			Test of heterogeneity		
		OR	95% CI	*P*-value	Model	*P*-value	I^2^(%)
rs7517847	G vs. T	0.99	0.84–1.16	0.87	R	0.08	51
	GG vs. GT + TT	0.59	0.31–1.13	0.11	R	0.0007	82
	GG + GT vs. TT	0.94	0.81–1.10	0.46	F	0.14	42
	GG vs. TT	0.84	0.59–1.22	0.36	R	0.03	61
rs17375018	A vs. G	0.64	0.40–1.04	0.07	R	<0.00001	90
	AA vs. AG + GG	0.76	0.43–1.33	0.34	R	0.05	61
	AA + AG vs. GG	0.54	0.28–1.04	0.06	R	<0.00001	91
	AA vs. GG	0.54	0.23–1.25	0.15	R	0.001	81
rs11209032	A vs. G	1.13	0.76–1.69	0.54	R	<0.0001	89
	AA vs. AG + GG	1.21	0.88–1.65	0.24	R	0.11	50
	AA + AG vs. GG	1.07	0.72–1.58	0.75	R	0.02	71
	AA vs. GG	1.19	0.69–2.06	0.52	R	0.009	74

*R* random effects model, *F* fixed effects model

### Heterogeneity and publication bias

The genotype distributions in the normal control groups were consistent with HWE in all studies. Some heterogeneity was found in the meta-analyses of the IL-23R polymorphisms. Given the small number of included studies, it was difficult to correlate the funnel plot, which is usually used to detect publication bias. However, Egger’s regression test showed no evidence of publication bias (Egger’s regression test *P* values >0.1).

## Discussion

In this study, we addressed the association between IL-23R polymorphisms and susceptibility to uveitis. Data from published studies were combined to evaluate the genetic associations between the most commonly studied polymorphisms of IL-23R, rs7517847, rs17375018, and rs11209032, and uveitis.

There was no significantly association between the rs7517847 polymorphism and uveitis. The rs17375018 polymorphism was also not associated with uveitis, except the AA + AG vs. GG genotype. Although results of the rs17375018 A allele, AA vs. AG + GG genotype, and AA vs. GG genotype showed no significant association with uveitis, the A allele tended to decrease uveitis (OR 0.68, 95% CI 0.47–0.99, *P* = 0.05; OR 0.76, 95% CI 0.49–1.18, *P* = 0.22; OR 0.56, 95% CI 0.29–1.09, *P* = 0.09). In the present meta-analysis, combined evidence demonstrated that the IL23R gene rs11209032 polymorphisms were not associated with uveitis. However, the rs11209032 AA genotype was significantly associated with uveitis. We found that the homozygote models, including the rs17375018 GG genotype and rs11209032 AA genotype, were significantly associated with uveitis.

Many theories on the etiology of Fuchs’ syndrome have been suggested including viral infection, toxoplasmosis, vascular abnormality, and autoimmunity. Some studies have shown that infections such as rubella virus and toxoplasma could lead to the development of Fuchs’ syndrome. However, other studies found that patients with Fuchs’ syndrome had high levels of proinflammatory cytokines, the pathology of the iris was rich in CD8+ lymphocytes, and the aqueous humor had abundant oligoclonal IgG production. These findings indicate that Fuchs’ syndrome has immunologic characteristics. Thus, we performed this meta-analysis with and without Fuchs’ syndrome. The results showed that there was no association between IL23R polymorphisms and uveitis.

Th17 cells may be relevant to human uveitis. Transfer of Th17 cells led to neutrophilic infiltrates, which is consistent with the cytokine and chemokine profile typical of the respective responses [16]. Th17 cells bind to proinflammatory cytokines such as IL-1, IL-6, transforming growth factor β (TGF-β), IL-21, and IL-23. IL-23 activity is mediated by its binding to the IL-23R on 17 helper T (Th17) cells. The IL23-Th17 interactions are thought to play an important role in the development of autoimmune disease [7]. IL-23 is a heterodimeric regulatory cytokine that is produced by activated macrophages and dendritic cells. IL-23 induces a cell population with a unique inflammatory gene signature. This signature includes IL-17A, IL-17F, IL-6, colony-stimulating factor 2 (CSF2), tumor necrosis factor (TNF), CC-chemokine ligand 20 (CCL20), CCL22, IL-1 receptor type 1 (IL-1R1), and IL-23R [17].

IL-23 was found to be elevated in VKH syndrome and Behçet’s disease [18, 19]. Some previous studies also showed an association between SNPs in IL-23R and uveitis. However, the results varied based on the accompanying systemic disease or cause of uveitis [5, 6]. This study analyzed the association between the rs7517847, rs17375018, and rs11209032 polymorphisms and uveitis. However, some studies showed significant associations between uveitis and other polymorphisms of IL-23R, such as rs924080and rs117633859 [20, 21].

The strength of our meta-analysis could be summarized as follows. We attempted to identify as many publications as possible using various search techniques. To the best of our knowledge, our study is the first meta-analysis regarding overall uveitis. It includes uveitis of ankylosing spondylitis, Behçet’s disease, VKH syndrome, sarcoidosis, Fuchs’ syndrome, and any other endogenous non anterior uveitis. All studies satisfied HWE. This implies that there was no potential bias regarding the control selection or genotyping errors.

This study also has several limitations. First, there was significant heterogeneity in some comparisons. There was variability in both diseases of developing uveitis as well as ethnicity. Unfortunately, stratification for ethnicity was not possible due to the low number of published studies on this topic. In addition, because only English publications were included, there may have been a language bias. A third limitation is that publication bias may have affected the analysis, because studies that produced negative results may not have been published or may have been missed. Fourth, we did not conduct a meta-analysis of haplotype analysis; however, haplotype analysis may have provided more information and would have been more powerful than single polymorphism analysis. Fifth, we did not classify the cases into anterior uveitis, posterior uveitis, and panuveitis. Lastly, although IL-23R polymorphisms may be associated with disease severity and susceptibility, we did not perform a meta-analysis for this association.

## Conclusions

The rs7519847, rs17375018 and rs11209032 alleles of IL-23R were negatively associated with uveitis. However, our results also suggest that both rs17375018 GG and rs11209032 alleles of IL-23R are predisposing genotypes for uveitis. These results should be interpreted with caution, given the study’s limitations. Further studies with larger sample sizes, as well as polymorphisms of other proinflammatory cytokines, are needed.

## Abbreviations

CCL20: CC-chemokine ligand 20
CI: Confidence interval
CSF2: Colony-stimulating factor 2
HWE: Hardy-Weinberg equilibrium
IL-1R1: IL-1 receptor type 1
IL-23R: Interleukin-23 receptor
OR: Odds ratio
SNPs: Single-nucleotide polymorphisms
TGF-β: Transforming growth factor β
Th17: 17 helper T
TNF: Tumor necrosis factor
VKH: Vogt-Koyanagi-Harada

## References

[1] Prete M, Guerriero S, Dammacco R, Fatone MC, Vacca A, Dammacco F (2014). Autoimmune uveitis: a retrospective analysis of 104 patients from a tertiary reference center. J Ophthalmic Inflamm Infect.

[2] Cenit MC, Marquez A, Cordero-Coma M, Gorrono-Echebarria MB, Fonollosa A, Adan A, et al. No evidence of association between common autoimmunity STAT4 and IL23R risk polymorphisms and non-anterior uveitis. PLoS One. 2013;8:e72892. 10.1371/journal.pone.0072892 PMC3843656 24312163

[3] Barisani-Asenbauer T, Maca SM, Mejdoubi L, Emminger W, Machold K, Auer H (2012). Uveitis- a rare disease often associated with systemic diseases and infections- a systematic review of 2619 patients. Orphanet J Rare Dis.

[4] Cho JH, Gregersen PK (2011). Genomics and the multifactorial nature of human autoimmune disease. N Engl J Med.

[5] Zhou H, Jiang Z, Yang P, Hou S, Li F, Shu Q (2010). Polymorphisms of IL23R and Fuchs’ syndrome in a Chinese Han population. Mol Vis.

[6] Jiang Z, Yang P, Hou S, Du L, Xie L, Zhou H (2010). IL-23R gene confers susceptibility to Behcet's disease in a Chinese Han population. Ann Rheum Dis.

[7] Kim HS, Choi D, Lim LL, Allada G, Smith JR, Austin CR (2011). Association of interleukin 23 receptor gene with sarcoidosis. Dis Markers.

[8] Dong H, Li Q, Zhang Y, Tan W, Jiang Z (2013). IL23R gene confers susceptibility to ankylosing spondylitis concomitant with uveitis in a Han Chinese population. PLoS One.

[9] Moher D, Liberati A, Tetzlaff J, Altman DG (2009). Preferred reporting items for systematic reviews and meta-analyses: the PRISMA statement. J Clin Epidemiol.

[10] Higgins JP, Thompson SG (2002). Quantifying heterogeneity in a meta-analysis. Stat Med.

[11] Egger M, Smith GD, Phillips AN (1997). Meta-analysis: principles and procedures. BMJ.

[12] DerSimonian R, Laird N (1986). Meta-analysis in clinical trials. Control Clin Trials.

[13] Egger M, Davey Smith G, Schneider M, Minder C (1997). Bias in meta-analysis detected by a simple, graphical test. BMJ.

[14] Gheita TA, Gamal SM, Shaker I, El Fishawy HS, El Sisi R, Shaker OG (2015). Clinical significance of serum interleukin-23 and a/G gene (rs17375018) polymorphism in Behcets disease: relation to neuro-Behcet, uveitis and disease activity. Joint Bone Spine.

[15] Kreps EO, Derveaux T, De Keyser F, Kestelyn P (2016). Fuchs’ Uveitis syndrome: no longer a syndrome?. Ocul Immunol Inflamm.

[16] Horai R, Caspi RR (2011). Cytokines in autoimmune uveitis. J Interf Cytokine Res.

[17] Gaffen SL, Jain R, Garg AV, Cua DJ (2014). The IL-23-IL-17 immune axis: from mechanisms to therapeutic testing. Nat Rev Immunol.

[18] Chi W, Yang P, Li B, Wu C, Jin H, Zhu X (2007). IL-23 promotes CD4+ T cells to produce IL-17 in Vogt-Koyanagi-Harada disease. J Allergy Clin Immunol.

[19] Chi W, Zhu X, Yang P, Liu X, Lin X, Zhou H (2008). Upregulated IL-23 and IL-17 in Behcet patients with active uveitis. Invest Ophthalmol Vis Sci.

[20] Cavus F, Ulusoy C, Orcen A, Gul A, Tuzun E, Vural B (2014). Increased IL-23 receptor, TNF-alpha and IL-6 expression in individuals with the IL23R-IL12RB2 locus polymorphism. Immunol Lett.

[21] Hou S, Du L, Lei B, Pang CP, Zhang M, Zhuang W (2014). Genome-wide association analysis of Vogt-Koyanagi-Harada syndrome identifies two new susceptibility loci at 1p31.2 and 10q21.3. Nat Genet.

